# Identification of novel B-cell epitope in gp85 of subgroup J avian leukosis virus and its application in diagnosis of disease

**DOI:** 10.1186/s12917-018-1622-x

**Published:** 2018-09-26

**Authors:** Kun Qian, Xue Tian, Hongxia Shao, Jianqiang Ye, Yongxiu Yao, Venugopal Nair, Aijian Qin

**Affiliations:** 1 Ministry of Education Key Lab for Avian Preventive Medicine, College of Veterinary Medicine, Yangzhou University, No.12 East Wenhui Road, Yangzhou, Jiangsu 225009 People’s Republic of China; 2 The International Joint Laboratory for Cooperation in Agriculture and Agricultural Product Safety, Ministry of Education, Yangzhou University, 225009 Yangzhou, People’s Republic of China; 3 Jiangsu Key Lab of Zoonosis, No.12 East Wenhui Road, Yangzhou, Jiangsu 225009 People’s Republic of China; 4 Avian Oncogenic Virus Group, The Pirbright Institute, Ash Road, Pirbright, Surrey, GU24 0NF UK; 5 The UK-China Centre of Excellence for Research on Avian Diseases, 169 Huanghe 2nd Road, Binzhou, Shandong People’s Republic of China

**Keywords:** Avian leukosis virus subgroup J, B-cell epitope mapping, Epitope-based peptide ELISA, Antibody detection

## Abstract

**Background:**

The gp85 is the main envelope protein of avian leukosis subgroup J (ALV-J) involved in virus neutralization. Here, we mapped the epitope in ALV-J gp85 by ELISA using synthetic peptides and developed epitope based diagnostic methods for ALV-J infection.

**Results:**

The results revealed that monoclonal antibody (mAb) JE9 recognized ^83^WDPQEL^88^ motif, which was highly conserved in gp85 among different ALV-J strains by homology analysis. Moreover, after evaluation with two hundred and forty sera samples obtained from different chicken farms, the epitope-based peptide ELISA had much higher sensitivity than commercial ELISA kit for antibody detection of ALV-J.

**Conclusions:**

A novel B-cell epitope recognized by the mAb JE9 was identified. The developed peptide-ELISA based on this novel B-cell epitope could be useful in laboratory viral diagnosis, routine surveillance in chicken farms, and also in understanding the pathogenesis of ALV-J.

## Background

Since the first report of avian leukosis virus subgroup J (ALV-J) in the United Kingdom in 1988, the virus has spread rapidly to many countries including China [1–5]. As the most prevalent subgroup currently in China, ALV-J infection causes vascular and visceral neoplasms, decrease egg production, stunted growth, and increased mortality [3, 6]. Compared to the Western countries where ALV-J-associated disease was restricted to the meat-type chickens, the disease in China was also seen in layer flocks and local chicken breeds, possibly due to the extremely high mutation rates [7, 8].

The genome of ALV-J consists of three viral structural genes, *gag*, *pol*, and *env*, which encode the group-specific (gs) antigen, integrase and reverse transcriptase, and envelop glycoprotein, respectively. The *env* gene encodes two proteins gp85 and gp37, which are synthesized as a single precursor polypeptide. The gp85 protein contains the determinants of ALV subgroup specificity, virus neutralization and receptor binding [9, 10]. Meanwhile, the gp85 is the most variable structural protein which exhibits high diversity in the genome of ALV-J [7, 11, 12]. Therefore, identification of the conserved epitopes in gp85 will facilitate the establishment of serological methods for the detection of ALV-J.

In our previous report, we showed that the gp85-specific mAb JE9 could react with different ALV-J strains but not with other ALV subgroups [13], confirming that the mAb JE9 recognized a conserved antigenic epitope. However, the exact epitope sequence recognised by the mAb JE9 has not been identified. In this study, we identified a conserved linear B-cell epitope recognised by the mAb JE9 using synthetic peptides, and applied it for the diagnosis of ALV-J infection using an epitope-based peptide ELISA. The results in this study will contribute to the understanding of the antigenic structure of gp85 and rational design of vaccines and diagnostic tools.

## Results

### Epitope mapping in gp85 protein recognized by mAb JE9

**Fig. 1:**
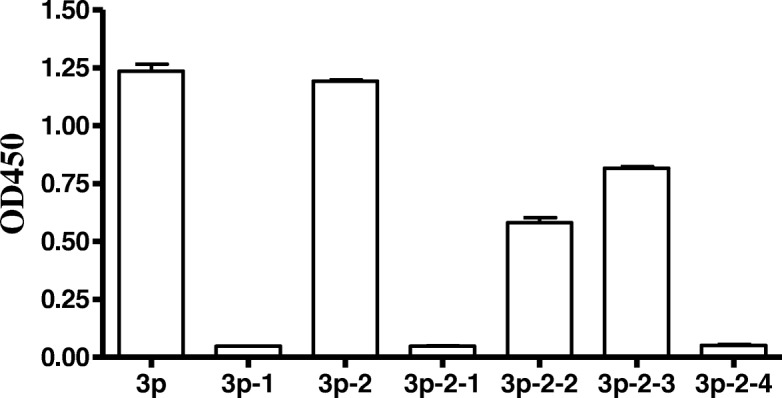
Reactivity of the different synthetic peptides of gp85 with mAb JE9 using ELISA. The name of each column corresponds to the polypeptide in the Table 2

Our preliminary unpublished data using western blot assay showed that the mAb JE9 recognized epitope between the amino acid positions 65 to 155 of gp85 protein. For precise mapping of this epitope, ALV-J-1P (95-125aa), ALV-J-2P (126-155aa) and ALV-J-3P (65-94aa), which covered 65–155 aa of gp85 protein were synthesized. The OD450 values of peptide ELISA revealed that JE9 reacted with ALV-J-3P but not with the other two (Table 1). Subsequently, ALV-J-3P was further truncated into different overlapping peptides described in Table 2. Accordingly, we identified WDPQEL as the target sequence of mAb JE9, which corresponds to 83–88 aa of ALV-gp85 as deletion of 83 W or 88 L disrupted the binding of the peptides with mAb JE9. The results indicated that the peptide ^83^WDPQEL^88^ was the minimal epitope in the gp85 protein of ALV-J for binding with mAb JE9 (Fig. 1, Table 2).

**Table 1:** Reactivity of the different synthetic peptides of gp85 with mAb JE9 using ELISA

	ALV-J-1P (95-125aa)	ALV-J-2P (126-155aa)	ALV-J-3P (65-94aa)
PBST	0.055^a^	0.047	0.054
JE9	0.049	0.056	1.236

^a^The mean values of triplicate OD450 detected by Bio-TEK ELISA reader

**Table 2:** Epitope mapping of mAb JE9 with synthetic peptides ELISA

Peptide ID	Sequence	Location	Reaction^a^
ALV-J-3p	DLASQTACLIQALNTTLPWDPQELDILGSQ	65–94	+
ALV-J-3p-1	DLASQTACLIQALNTTLPWD	65–84	–
ALV-J-3p-2	QALNTTLPWDPQELDILGSQ	75–94	+
ALV-J-3p-2-1	QALNTTLPWDPQE	75–87	–
ALV-J-3p-2-2	WDPQELDILGSQ	83–94	+
ALV-J-3p-2-3	QALNTTLPWDPQEL	75–88	+
ALV-J-3p-2-4	DPQELDILGSQ	84–94	–

^a^Reaction of peptide coated ELISA with mAb JE9

### The epitope is conserved among ALV-J strains

**Fig. 2:**
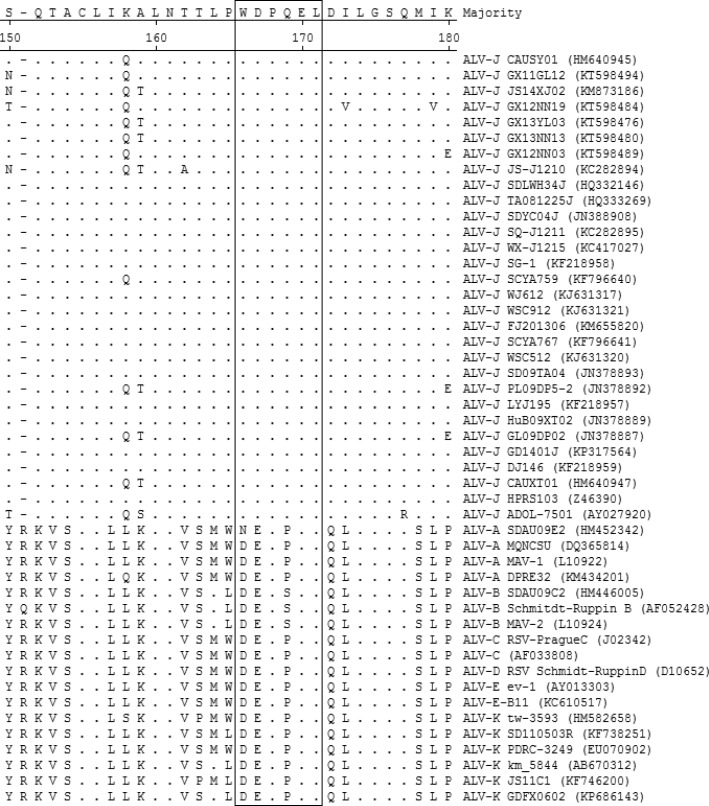
Alignment of the epitopes motif with 51 ALV strains. The GenBank accession numbers of the ALV strains used are indicated in parentheses. The homologous sequences of different ALV strains corresponding to the identified epitope are boxed. Identical residues are indicated by “.”. “-” indicates that there was no corresponding amino acid at this position

In order to evaluate the conservation of the mAb JE9 defined epitope, alignment analysis was performed with gp85 sequences of 25 ALV-J strains, 6 ALV-A strains, 6 ALV-B strains, 2 ALV-C strains, 1 ALV-D strain, 5 ALV-E strains, and 6 ALV-K strains reported in recent year. As illustrated in Fig. 2, the alignment results showed that the ^83^WDPQEL^88^ is highly conserved among all ALV-J strains analysed.

### Development and optimization of the peptide-ELISA procedure

In order to achieve the best reactivity of the peptide-ELISA, according to the previous experimental results and cost considerations, we choose BSA conjugated polypeptide ALV-J-3P-2 (BSA-3P-2), BSA –C-^75^QALNTTLPWDPQELDILGSQ^94^, as ELISA coating antigen. The optimal dilution of reagents was determined at 1 μg/ml for BSA-3P-2 peptide, 8% Normal Rabbit Serum in PBS with 0.05% Tween 20 for blocking reagent, 1:200 for serum sample and 1:10000 for the secondary horseradish peroxidase-conjugated rabbit anti-chicken immunoglobulin. Exposure time was optimally at 37 °C for 25 min. Using these conditions, the best signal with minimum background was obtained in the peptide-ELISA. And the cross reaction of the peptide-ELISA was excluded by sera from other common avian diseases (data not shown).

### Determination of the cut-off value

One hundred and seventy SPF serum samples were tested by peptide-ELISA to set cut-off values. The 170 SPF serum samples had mean OD_450_ of 0.087 ± 0.022. Thus, for higher specificity the cut-off value for this peptide-ELISA could be determined at 0.153 by adding three standard deviations to the mean for SPF serum of 0.087. For the test system to be valid, we determined that the positive control OD_450_ should be higher than 0.4 and at least three times higher than the negative control OD_450_. And the negative control OD_450_ should be lower than mean plus 2 standard deviations, 0.131. If the values of positive or negative control were outside these limits, the test was repeated.

### Repeatability and reproducibility of the peptide-ELISA

Results from Table 3 determined the repeatability and reproducibility of the diagnostic assay by seven sera samples. The intraplate variation showed CVs from 3.34 to 7.81% (4.89% average), whereas the interplate variation showed CVs from 2.74 to 7.30% (4.14% average). The good reflection of assay precision was proved by the low calculated CV values.

**Table 3:** Intraplate and interplate variation of the peptide-ELISA

Sample No.	1	2	3	4	5	6	7
Intraplate CV (%)	3.58	5.81	3.34	3.67	4.20	7.81	5.81
Interplate CV (%)	2.74	4.06	2.79	2.88	4.13	7.30	5.07

### Application of peptide-ELISA to test field samples and comparison with commercial ELISA

**Fig. 3:**
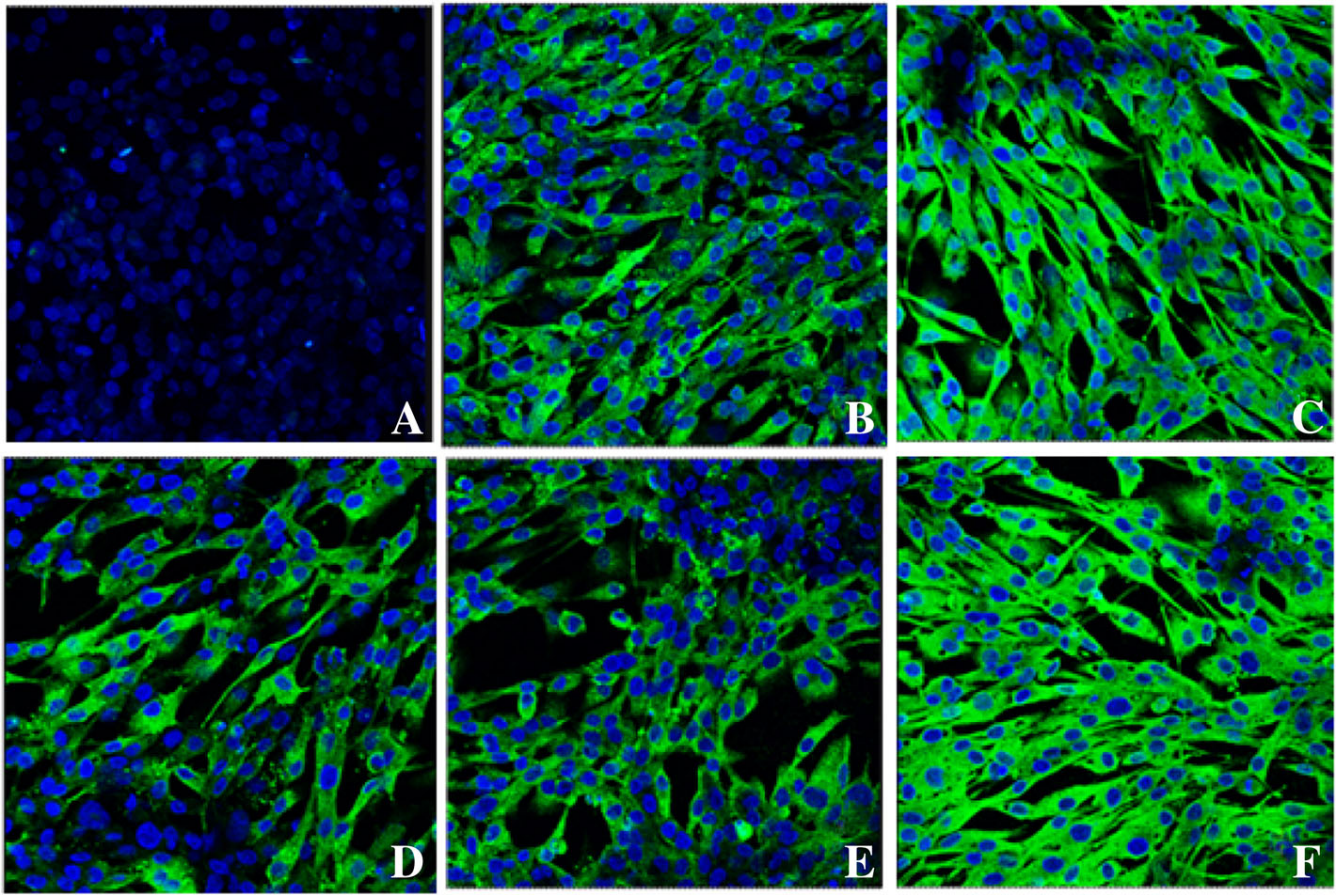
The result of partial serum samples evaluated with IFA. Panel **a** and **b** were negative and positive control respectively; Panel **c** to **f** were partial positive results by serum samples which were negative in IDEXX ELISA, but positive in peptide-ELISA

To validate the performance of the peptide-ELISA, 240 sera samples derived from different chicken farms were detected by peptide-ELISA and IDEXX ELISA, and IFA served as gold standard method to evaluate the results detected by these two ELISAs. As shown in Table 4, the positivity of peptide-ELISA was 20.42% (49/240), as compared with 4.58% (11/240) for IDEXX ELISA. The 11 positive samples from IDEXX ELISA were all included in the positive results of peptide-ELISA. When the IFA was used as the standard for comparison, we found that the sensitivity of peptide-ELISA and IDEXX ELISA was 85.96% (49/57) and 19.30% (11/57). The specificity of two methods was 95.63% (175/183) and 100% (183/183) respectively (Table 4). The results clearly showed that the peptide-ELISA for ALV-J antibody detection developed in this study had much more sensitivity than commercial ELISA when applied to field sera samples. In order to further verify the specificity of 38 sera samples which were negative in IDEXX ELISA, the IFA was carried out with these sera as primary antibodies. The results in Fig. 3 showed 29 out of 38 sera samples were positive in IFA assay.

**Table 4:** Evaluation of the developed peptide-ELISA for detection ALV-J antibodies with clinical serum samples

		Peptide-ELISA		IDEXX ELISA^a^	
		Positive	Negative	Positive	Negative
IFA	Positive	49	8	11	46
	Negative	8	175	0	183

^a^ALV-J antibody ELISA kit obtained from IDEXX Inc., Beijing China

## Discussion

Invention and development of monoclonal antibody technology provide an easy approach to identify the B-cell epitope which can induce antibody production. The mAb JE9 used in current study is the earliest reported monoclonal antibody specific to the gp85 protein of ALV-J. According to previous reports, the mAb JE9 has good reactivity with many ALV-J isolates all over the world [13]. It suggests that the B-cell epitope recognized by JE9 is a conserved and immunodominant epitope. The results of peptide epitope mapping and homology analysis in Fig. 1 and Fig. 2 proved that the ^83^WDPQEL^88^ peptide sequence is highly conserved and specific among ALV-J strains.

The peptide-ELISA has been shown to be a sensitive and specific indirect diagnostic tool in virology of a multitude of species, ranging from humans in public health to livestock in the agriculture industry [14–18]. How to choose the peptide sequence is the key point to the peptide-ELISA. The binding ability of the selected polypeptide to the antibody, the length of the polypeptide and the necessity to be conjugated with the carrier are factors that need to be considered. In the results of Fig. 1, when we truncated the 3p-2 peptide to ^83^WDPQELDILGSQ^94^ and ^75^QALNTTLPWDPQEL^88^ which contain the minimal conserved epitope, the reactivity of the polypeptide decreased significantly although the OD_450_ value was still positive. The similar results have been reported in previous study, which described that peptide length alters diagnostic sensitivity and specificity [15]. In the same report, the authors suggested that conjugation of peptides to BSA could improve assay sensitivity. In current study, we got the similar results also. When the peptide was conjugated with BSA, the reactivity of the peptide-ELISA slightly enhanced (data not shown). In another report, B-cell epitope for ALV-J gp85 protein, ^134^AEAELRDFI^142^, was identified [19]. We combined ALV-J-2P which covered 134-142aa and ALV-J-3P-2 in the same ELISA. Unfortunately, the sensitivity was not improved, but on the contrary the reactivity decreased (data not shown). Thus, we choose BSA conjugated 3p-2 only as the coating antigen in the peptide-ELISA for ALV-J antibody detection.

Full length proteins are normally effective reagents for immunodiagnostics because they contain multiple epitopes. However, in this study the peptide-ELISA for ALV-J antibody detection has much more sensitivity than IDEXX ELISA kit which coated with the whole gp85 of ALV-J expressed by recombinant baculovirus. We speculate that glycosylated gp85 protein expressed by recombinant virus might block the internal antigenic epitope which can be recognized by the positive sera. In addition, there are nine sera samples positive in peptide-ELISA, but negative in IFA assay. This suggests more positive and negative samples need to be tested to revise cut-off value to make the ELISA more precise in the future.

## Conclusions

In summary, a novel B-cell epitope, ^83^WDPQEL^88^, specific for ALV-J gp85 protein recognized by the mAb JE9 was identified in this study. The peptide-ELISA based on this novel B-cell epitope established here was more sensitive for ALV-J antibody than current serological method for ALV-J in IDEXX ELISA. The successful use of this peptide in detecting ALV-J in the clinical serum samples suggests that the epitope-based peptide ELISA could possibly be used as a serologic reagent in the diagnosis of ALV-J infection and it will contribute to the rational design of vaccines by further understanding of the antigenic structure of gp85.

## Methods

### Cells, antibody and ELISA kit

The pcDNA-env-DF1 cell line [10] were maintained in Dulbecco’s modified Eagle medium (DMEM; GIBCO, Shanghai China) supplemented with 5% fatal bovine serum (FBS), 100 U/mL of penicillin, 100 g/mL of streptomycin and 100 μg/mL of Zeocin at 37 °C in a 5% CO_2_ atmosphere. ALV-J gp85 specific monoclonal antibody, JE9, was kept in our lab [13]. The antibodies of chicken sera samples were determined by ALV-J antibody ELISA (IDEXX, Beijing, China) according to the manufacturer’s protocol.

### Sera samples collection

The 170 SPF chicken sera samples were kindly provided by Spirax Ferrer Poultry Science and Technology Co.Ltd., Jinan, China. The other 240 chicken sera samples were collected from chicken farms with the eradication programme of ALV being conducted in Jiangsu and Shandong Province in China. All experiments complied with institutional animal care guidelines and were approved by the Animal Care Committee of Yangzhou University.

### Peptide design and synthesis

The 65–155 amino acids of gp85 protein were equally divided into three segments, ALV-J-1P (95-125aa), ALV-J-2P (126-155aa), and ALV-J-3P (65-94aa), respectively. A series of peptides of ALV-J-3P for mAb epitope mapping was designed in Table 2. All of the peptides were synthesized by Shanghai Jietai Biotech Company. The crude peptides were purified by semi-preparative HPLC on a Beckman System Gold with a reverse-phase C18 column, resulting in purity greater than 95%, checked by analytical HPLC on a Shimadzu system. The BSA conjugated polypeptide (BSA-3P-2) was also synthesized and purified by Shanghai Jietai Biotech Company.

### Homology analysis

The conservation of the sequence that contained the B-cell epitope of gp85 protein was analysed with sequences of different ALV strains using MegAlign software version 7.1.0 (DNAstar, Madison, USA). The sequences of the ALV strains that were used as reference were downloaded from the GenBank database. Analysis of the antigenic index and the surface probability of the ALV gp85 protein was also performed using Protean software version 7.1.0 (DNAstar, Madison, USA).

### Peptide-ELISA procedure

The peptide-ELISA procedure was performed as previously described with minor modifications [17]. Maxisorp ELISA plates (NUNC, Thermo, Shanghai) were coated overnight at 4 °C with 100 μl per well of a 1 μg/ml peptide solution in 0.1 M carbonate/bicarbonate buffer, pH 9.6. Next morning, 400 μl per well of 8% Normal Rabbit Serum (Biyuntian, Nantong, China) in PBS was added for blocking, and the plate was incubated for 2 h at 37 °C followed by three washes with PBS supplemented with 0.05% Tween 20. Reagent layers were removed by striking plates repeatedly, bottom up, on a stack of absorbent tissue.

After blocking and washing, the plate was incubated with 100 μl per well of chicken sera diluted in 8% Normal Rabbit Serum in PBS with 0.05% Tween 20 at 37 °C for 30 min. Unless stated otherwise, sera were assayed at a 1:200 dilution. After being washed, plates were incubated with 100 μl per well of horseradish peroxidase-conjugated rabbit anti-chicken immunoglobulin (SIGMA, Shanghai, China) at 37 °C for 45 min, diluted 1:10000 in 8% Normal Rabbit Serum in PBS with 0.05% Tween 20. Following a final wash, plates were developed for approximately 25 min at 37 °C with 100 μl per well of TMB Turbo substrate (Pierce, Thermo, USA), and were stopped with 100 μl per well of 2 M sulphuric acid. Absorbance at OD450 was determined using a standard ELISA plate reader (Bio-TEK, Vermont, USA).

For storage, ELISA plates were coated with peptide and blocked as described above. Excess blocking reagent was removed, and the plates were dried with bottom up and the wells exposed to circulating air, for 4 h at room temperature. Plates were then stored in Vacuum packaging at 4 °C. The ELISA protocol for pre-coated plates was the same as described above, starting with the addition of the primary antibody.

### Preliminary precision assessment

Preliminary precision assessment was performed as previously described [20]. Coefficients of variation (CVs) for 3 replicates of a total of 7 specimens were run on the same plate and calculated for repeatability (intraplate variation). For reproducibility, CVs were obtained by using the same specimens run in triplicate in two different plates (interplate variation).

### Indirect immunofluorescence assay (IFA)

The protocol was the same as that of our previous report [21]. Briefly, the pcDNA-env-DF1 cells were fixed with 4% paraformaldehyde in PBS for 20 min at room temperature, permeabilized with 0.25% Triton X-100 for 5 min, washed with PBS, blocked with 2% BSA for 30 min, and incubated with chicken serum (1:20 dilution) in PBS for 45 min at room temperature. The cells were then washed in PBS and incubated with rabbit anti-chicken IgG conjugated with FITC (Sigma, Shanghai, China) and stained with 10 μg/mL of Hoechst 33342 dye (SIGMA, Shanghai, China) at room temperature for an additional 30 min. The pictures were captured with a Leica SP2 confocal microscope.

### Statistical analysis

Statistical analysis including CV calculation was performed by using GraphPad Prism version 5.0 software (GraphPad Software, SanDiego, CA).

## Abbreviations

ALV-J: Avian leukosis virus subgroup J
BSA: Bovine serum albumin
DMEM: Dulbecco’s modified Eagle’s medium
ELISA: Enzyme-linked immunosorbent assay
*env*: envelop;
FBS: Foetal bovine serum
FITC: Fluorescein isothiocyanate
mAb: Monoclonal antibody
